# Effect of Electro-Thermo-Mechanical Coupling Stress on Top-Cooled E-Mode AlGaN/GaN HEMT

**DOI:** 10.3390/ma16041484

**Published:** 2023-02-10

**Authors:** Jie Jiang, Qiuqi Chen, Shengdong Hu, Yijun Shi, Zhiyuan He, Yun Huang, Caixin Hui, Yiqiang Chen, Hao Wu, Guoguang Lu

**Affiliations:** 1 School of Microelectronics and Communication Engineering, Chongqing University, Chongqing 400044, China; 2 The Science and Technology on Reliability Physics and Application of Electronic Component Laboratory, China Electronic Product Reliability and Environmental Testing Research Institute, Guangzhou 510610, China; 3 Science and Technology on Analog Integrated Circuit Laboratory, Chongqing 401332, China

**Keywords:** AlGaN/GaN, HEMT, electro-thermo-mechanical coupling, COMSOL

## Abstract

This work investigated the effects of single stress and electro-thermo-mechanical coupling stress on the electrical properties of top-cooled enhancement mode (E-mode) Aluminium Gallium Nitride/Gallium Nitride (AlGaN/GaN) high electron mobility transistor (HEMT) (GS66508T). Planar pressure, linear deformation, punctate deformation, environmental temperature, electro-thermal coupling, thermo-mechanical coupling, and electro-thermo-mechanical coupling stresses were applied to the device. It was found that different kinds of stress had different influence mechanisms on the device. Namely, excessive mechanical pressure/deformation stress caused serious, irrecoverable degradation of the device’s leakage current, with the gate leakage current (*I_g_*) increasing by ~10^7^ times and the drain-to-source leakage current (*I_dss_*) increasing by ~10^6^ times after mechanical punctate deformation of 0.5 mm. The device characteristics were not restored after the mechanical stress was removed. Compared with three mechanical stresses, environmental thermal stress had a greater influence on the device’s transfer characteristic and on-resistance (*R_on_*) but far less influence on *I_g_* and *I_dss_*. As was expected, multiple stress coupled to the device promoted invalidation of the device. For more in-depth investigation, finite element simulation carried out with COMSOL was used to analyze the effect of electro-thermo-mechanical coupling stress on top-cooled E-mode AlGaN/GaN HEMT. The results of the experiments and simulation demonstrated that single and coupled stresses, especially mechanical stress coupled with other stresses, degraded the electrical properties or even caused irreversible damage to top-cooled E-mode AlGaN/GaN HEMT. Mechanical stress should be reduced as much as possible in the packaging design, transportation, storage, and application of top-cooled E-mode AlGaN/GaN HEMT.

## 1. Introduction

With the rapid development of power electronics technology, the performance of silicon-based power devices has approached its theoretical material limit. As a representative third-generation semiconductor material, gallium nitride (GaN) is widely used in devices in high power, high temperature, and high frequency fields of power electronics because of its superior material characteristics, such as high electron saturation speed, high electron mobility, high critical electric field, wide band gap, and superior thermal conductivity [1,2,3]. GaN-based high electron mobility transistors (HEMTs) are favored because of their mature process [4,5,6]. However, strong polarization between Aluminium Gallium Nitride (AlGaN) and GaN will confine electrons to the surface of the gallium nitride channel, thus forming a two-dimensional electron gas (2DEG) with high electron mobility and density [7,8,9], making GaN-based HEMTs normally-on devices. For the requirements of fail-safe operation, normally-off GaN HEMTs are highly preferred in power conversion systems and can be achieved by gate recess, p-GaN gate, fluorine plasma ion implantation, and tri-gate design, among which p-GaN HEMTs have recently been commercialized.

For p-GaN HEMTs to obtain widespread usage, reliability is one of the crucial factors to be addressed [10,11,12,13,14,15,16,17,18,19]. Although there are many studies reporting the reliability of GaN-based HEMTs, such as the high thermal reverse bias test [10,11,12], high temperature gate bias stress-induced instability [13,14,15], hard switching robustness [16], and short-circuit safe operating area [17,18,19], the degradation or failure induced by mechanical pressure/deformation stress still need to be comprehensively studied. Mechanical pressure/deformation stress environments and even environments in which mechanical stress couples with other stresses occur during the transportation, storage, and application of p-GaN HEMTs [20]. 2DEG at the interface of the AlGaN/GaN heterostructure is sensitive to stress. The dependence of 2DEG concentration and distribution corresponding to the piezoelectric effect has been reported [21,22]. Additionally, the dependence of electron mobility on strain has also been reported [23,24]. However, no systematic studies exploring the influence of mechanical pressure/deformation stress and mechanical stress coupling other stresses on the electrical properties of p-GaN HEMTs have been reported, which is necessary for the device to obtain widespread usage.

This work investigates the effect of single stress and electro-thermo-mechanical coupling stress on the electrical properties of top-cooled enhancement mode (E-mode) AlGaN/GaN HEMT. This paper is arranged as follows. The first chapter introduces the research background. The second chapter introduces the experimental and simulation schemes. The experimental and simulation results under different stress conditions are presented and compared in the third chapter. Finally, the last chapter presents the conclusion.

## 2. Experimentation and Simulation

**Figure 1:**
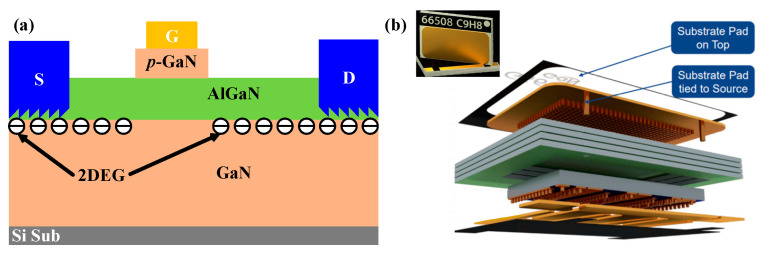
Schematic structure (**a**) and exploded view of package structure (**b**) of p-GaN HEMT (GS66508T) [20].

In this work, the GaN system’s E-mode AlGaN/GaN HEMT (Figure 1) with top-cooled pad (GS66508T) was used to carry out the electro-thermo-mechanical coupling experiments [20]. The top side of the device was partially covered with copper sheet, which was also connected to the internal chip’s substrate, as shown in Figure 1b. When the environmental thermal stress or the mechanical stress was applied to the top side of the device, the copper sheet transferred the corresponding stress to the inside chip, which caused changes in the electrical properties of the device.

In this research, planar pressure stress, linear deformation stress, punctate deformation stress, environmental temperature stress, electro-thermal coupling stress, thermo-mechanical coupling stress, and electro-thermo-mechanical coupling stress were applied to the device. Agilent B1500A was used to characterize the device’s transfer characteristic, on-resistance (*R_on_*), gate leakage current (*I_g_*), drain-to-source leakage current (*I_dss_*), and gate-lag characteristics before and after the stress experiments.

### 2.1. The Setup of the Electro-Thermo-Mechanical Experiment

The technical manual of GS66508T states that the package should be clamped to the heat sink when the device is operating, which will cause planar pressure. Pressure and deformation tests were described in another technical manual [20]. This indicated that mechanical pressure/deformation is very likely to be encountered not only when the device is working, but also in other situations, such as during package design, transportation, and storage of the device. There are also mechanical pressure/deformation stress environments, such as flat or irregular bumps and squeezing, or even environments in which mechanical stress couples with other stresses, such as high temperature environments, which may cause irreversible damage to the device when certain conditions are reached. In this study, the degradation behavior of the device was studied under three extreme mechanical stresses as well as under planar pressure coupled with other stresses.

#### 2.1.1. Mechanical Planar Pressure, Linear/Punctate Deformation Experiment

**Figure 2:**
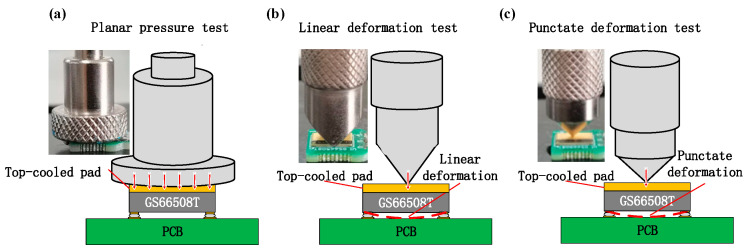
Schematic diagram and physical picture of the experimentally applied planar pressure (**a**), linear deformation (**b**), and punctate deformation (**c**) to GS66508T.

In Figure 2, the schematic diagrams and physical pictures of the experimentally applied pressure and deformation to top-cooled E-mode AlGaN/GaN HEMT (GS66508T) are shown. Three different mechanical pressure/deformation conditions were applied to GaN HEMT (GS66508T) through three pressure sensors. A planar pressure sensor is shown in Figure 2a, which can apply uniform planar pressure to cover the entire top copper sheet of the device. Figure 2b shows a sensor with two symmetrical oblique sections, which can apply linear pressure to the centerline of the top copper sheet. Compared with the planar pressure sensor, the contact area between the device and sensor in Figure 2b is smaller. Under the same amount of pressure, the pressure intensity of the sensor in Figure 2b is much higher. Since the pressure is only applied to the centerline of the top copper sheet, linear deformation of the device is easily achieved. Figure 2c shows a sensor with a sharp point, which can apply punctate pressure to the midpoint of the top copper sheet. Compared with the other sensors, the sensor in Figure 2c has the smallest contact area and largest pressure intensity under the same amount of pressure, which easily produces the most serious punctate deformation.

#### 2.1.2. Environmental Temperature Experiment

Different constant external environmental temperatures were set to investigate the influence of environmental temperature stress on the electrical properties of top-cooled E-mode AlGaN/GaN HEMT.

#### 2.1.3. Electro-Thermal Coupling Experiment

The drain current (*I_d_*) was applied to the device to generate an electric self-heating effect and investigate the influence of electro-thermal coupling stress on the electrical properties of top-cooled E-mode AlGaN/GaN HEMT. *V_G_* = 5 V was set to keep GS66508T on and constant current *I_DS_* = 5 A was applied between the drain and source to generate electrical self-heating coupling in the device, and the heat was transferred to the top-cooled copper pad of GS66508T. A PT100 platinum resistance with 304 stainless steel housing was placed against the top of GS66508T to measure the temperature of the copper sheet. Temperature control module AI-526AG and solid-state relay SSR-25DD were used to control whether the gate power supply made the device on and generated heat, so as to maintain the constant preset temperature.

#### 2.1.4. Thermo-Mechanical Coupling Experiment

On the basis of the environmental temperature experiment, different mechanical planar pressures were superimposed to realize the thermo-mechanical coupling stress experiment.

#### 2.1.5. Electro-Thermo-Mechanical Coupling Experiment

On the basis of the electro-thermal coupling experiment, different mechanical planar pressures were superimposed to realize the electro-thermo-mechanical coupling experiment.

### 2.2. Electro-Thermo-Mechanical Coupling Simulation Model

**Figure 3:**
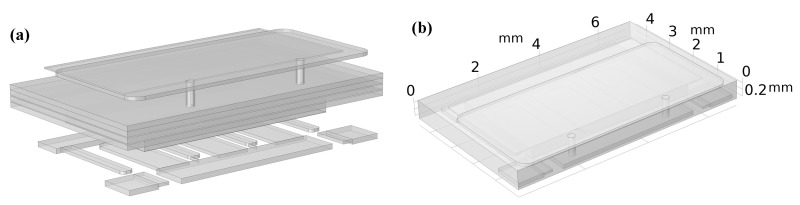
Exploded view of COMSOL simulation model (**a**) and top view of simulation model (**b**).

For more in-depth investigation, finite element simulation carried out with COMSOL was used to analyze the effect of electro-thermo-mechanical coupling stress on top-cooled E-mode AlGaN/GaN HEMT. The multi-field coupling simulation model constructed according to the actual structure of GS66508T is shown in Figure 3. Figure 3a shows the exploded view of the COMSOL simulation model of GS66508T established with reference to Figure 1b. Figure 3b shows the overall view of the COMSOL simulation model of GS66508T. The dimensions, location, and material of each part of the model are listed in Table 1, which is given approximately with reference to Figure 1b and the device data sheet [20].

## 3. Results and Discussion

### 3.1. Experimental Result and Discussion

In this work, 10 devices were measured in the mechanical planar pressure experiment, and their leakage currents (*I_g_* and *I_dss_*) were all significantly increased. After finding this phenomenon, we carried out further experimental studies and mainly observed changes in leakage currents. Additionally, more than 5 devices were also subjected to other experiments. In the planar pressure, linear deformation, punctate deformation, environmental temperature, and thermo-mechanical coupling experiments, the device was measured after stress had been applied and stabilized. In the electro-thermal coupling and electro-thermo-mechanical coupling experiments, the self-heating channel was immediately switched to the measured channel for measurement when the temperature reached a specified value, while other conditions remain unchanged.

#### 3.1.1. Mechanical Stresses

**Figure 4:**
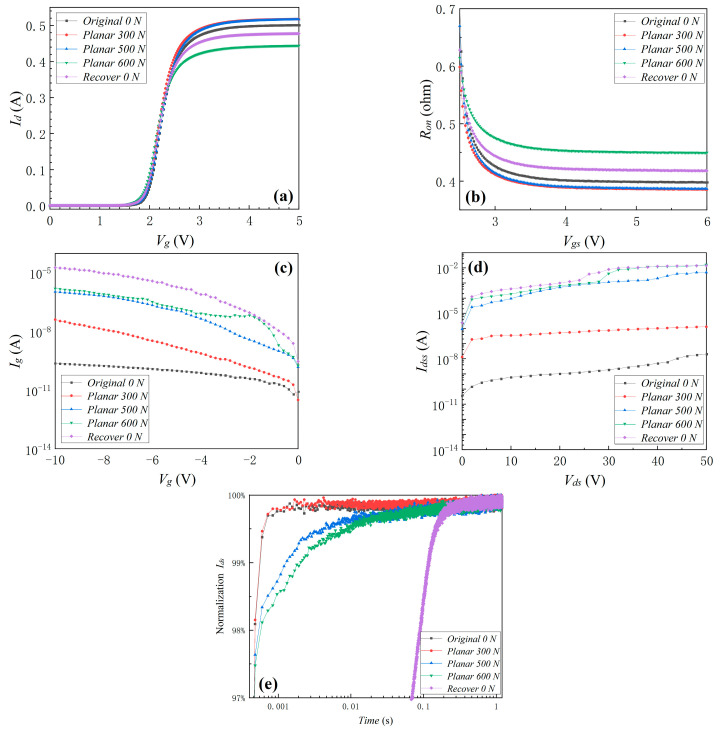
The device’s transfer characteristic (**a**), *R_on_* (**b**), *I_g_* (**c**), *I_dss_* (**d**), and gate-lag characteristics (**e**) before and after planar pressure of 300 and 500 N and recovery to 0 N.

For the sake of concise analysis, this section only shows the device’s electrical properties before and after planar pressure stress of 300, 500, and 600 N and recovery to 0 N (in this work, recovery means testing the device after removing all the stresses applied to the device), linear deformation stress of 0, 0.2 (pressure of 77 N), and 0.5 mm (pressure of 203 N) and recovery to 0 mm, punctate deformation stress of 0, 0.2 (pressure of 53 N), and 0.5 mm (pressure of 167 N) and recovery to 0 mm. Figure 4 shows the changes in the device’s transfer characteristic, *R_on_*, *I_g_*, *I_dss_*, and gate-lag characteristics before and after planar pressure stress. It can be seen that the threshold voltage (*V_th_*) did not exhibit obvious change with the increase in planar pressure stress. This indicated that the planar pressure stress did not affect the interface trapped charge of the device. *R_on_* decreased by 3.3% and 3.0% when the planar pressure stress increased from 0 to 300 and 500 N, respectively, but *Ron* increased by 12.8% when the planar pressure stress reached 600 N. These phenomena were due to tighter contact between the device pins and the printed circuit board (PCB) solder joints as the planar pressure increased, resulting in a decrease in contact resistance. At the same time, the new electron traps produced when excessive pressure was applied increased the negative charges, thus increasing the on-resistance of the device and subsequently decreasing the device’s on-state current. The change in contact resistance dominated when the pressure was below a certain threshold, and the new electron traps generated by the pressure dominated when it was greater than the threshold. Meanwhile, *I_g_* and *I_dss_* exhibited the most significant change with the increase in planar pressure stress, as shown in Figure 4c,d. After applied planar pressure stress of 300, 500, and 600 N, *I_g_* under *V_g_* = −10 V was 41.4 nA, 1.08 µA, and 1.58 µA, respectively. Compared with *I_g_* = 237 pA at 0 N, *I_g_* was increased by 100×, 4000×, and 6000×, respectively. The gate leakage current was increased by several orders of magnitude, indicating significant damage and degradation of the gate oxide layer during planar pressure application. In addition, after applied planar pressure stress, *I_dss_* under *V_ds_* = 50 V was 1.29 µA, 5.01 mA, and 16.0 mA, respectively. Compared with *I_dss_* = 20.5 nA at 0 N, *I_dss_* was increased by 60×, 200,000×, and 700,000×, respectively. The drain-to-source leakage current was increased by several orders of magnitude, indicating that the blocking characteristics of the device were significantly degraded during planar pressure application. The increase in both leakage currents may have been due to the new structural defects resulting from the planar pressure stress, which can form new leakage channels. After applied planar pressure stress of 0, 300, 500, and 600 N, the rise-time (the time of *I_d_* rise to 99% of the maximum current) was 600 µs, 600 µs, 1.32 ms, and 2.04 ms, respectively, as shown in Figure 4e. When the planar pressure reached 500 and 600 N, the rise-time increased significantly, indicating that the switching characteristics of the GaN device had degraded and the new electron traps of the GaN device had significantly increased, which explained the previous phenomena in Figure 4a–d. Then, after the pressure recovered to 0 N, the increase in *R_on_* was restored from 12.7% under 600 N to 4.7%, as shown in Figure 4b. However, *I_g_*, *I_dss_*_,_ and the rise-time had by no means recovered and degraded even more severely. The gate-lag characteristic indicated that when the pressure applied to the device was removed, new structural defects continued to grow, which formed new leakage channels, thus allowing both leakage currents to increase. This phenomenon indicated that the device had been irreversibly damaged under excessive planar pressure.

**Figure 5:**
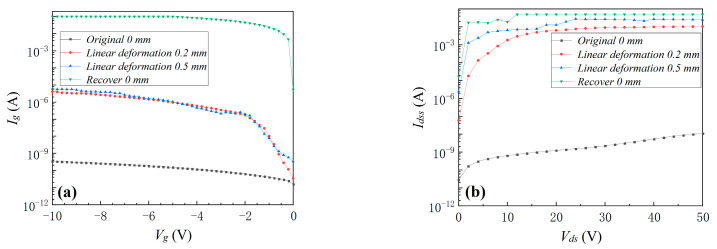
The device’s *I_g_* (**a**) and *I_dss_* (**b**) before/after linear deformation of 0.2 and 0.5 mm and recovery to 0 mm.

**Figure 6:**
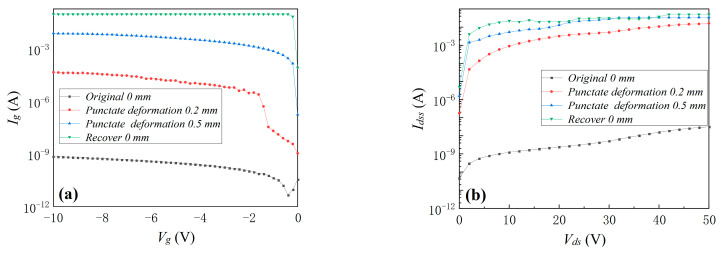
The device’s *I_g_* (**a**) and *I_dss_* (**b**) before/after punctate deformation of 0.2 and 0.5 mm and recovery to 0.

Because *I_g_* and *I_dss_* exhibited the most obvious changes under planar pressure stress, only *I_g_* and *I_dss_* before and after linear deformation and punctate deformation stresses will be compared in the following part. As shown in Figure 5 and Figure 6, when the linear deformation stress and punctate deformation stress increased, *I_g_* and *I_dss_* significantly increased. It can be seen from Figure 4, Figure 5 and Figure 6 that excessive mechanical pressure/deformation stress caused serious degradation of the device, and the device continued to degrade even after the pressure was removed. Comparing the degradation phenomena under three kinds of mechanical stresses revealed that the degradation due to mechanical linear/punctate deformation stress was more serious than that under planar pressure stress. To achieve the same deformation size, the pressure size required by punctate deformation was smaller than that of linear deformation, but the degradation was more severe. The smaller the contact area between GS66508T and the external mechanical stress source, the more electrical property degradation will take place.

In order to observe the obvious degradation trend and damage phenomenon, we applied mechanical stresses beyond the normal allowable values of GS66508T. It is possible that this situation could occur in mechanical pressure/deformation stress environments, such as flat or irregular bumps and squeezing during the transportation, storage, and application of GS66508T. From the previous mechanical stress experiments, it was clear that a flat object with a mass of 30 kg or a sharp object with a mass of 5 kg pressed against the device at rest could easily cause severe degradation and irrecoverable damage to the device’s performance. If the device or external object is in motion, less mechanical stress will be required for severe device degradation.

#### 3.1.2. Environmental Temperature Stress

**Figure 7:**
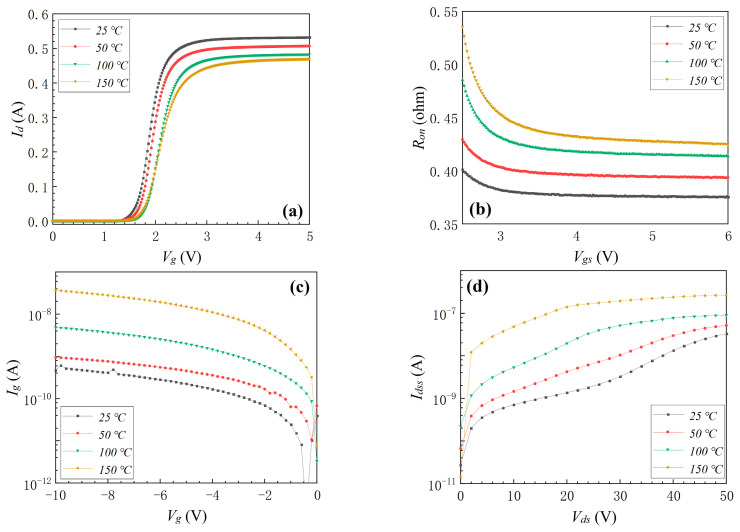
The device’s transfer characteristic (**a**), *R_on_* (**b**), *I_g_* (**c**), and *I_dss_* (**d**) at environmental temperatures of 25, 50, 100, and 150 °C.

The device’s transfer characteristic, *R_on_*, *I_g_*, and *I_dss_* at environmental temperatures of 25, 50, 100, and 150 °C are shown in Figure 7. When the environmental temperature increased from 25 to 150 °C, *V_th_* positive deviation gradually increased to 0.3 V while *R_on_* increased by 13.1%, which was due to the decrease in electron mobility caused by the increase in environmental temperature. The device’s *I_g_* and *I_dss_* increased when the environmental temperature increased from 25 to 150 °C, as shown in Figure 7c,d. *I_g_* under *V_g_* = −10 V was 952 pA, 4.88 nA, and 36.9 nA under temperatures of 50, 100, and 150 °C, respectively. The increase in the device’s *I_g_* with the environmental temperature was due to the increased probability of electron tunneling in the gate region with the increase in temperature. And device’s *I_dss_* was 51.7, 91.0, and 266 nA under temperatures of 50, 100, and 150 °C, respectively. The increase in the device’s *I_dss_* may have resulted from the increase in the leakage in buffer layer. It was found that compared with mechanical stresses, environmental thermal stress had a greater influence on the transfer characteristic and *R_on_* but far less influence on the device’s *I_g_* and *I_dss_*.

#### 3.1.3. Electro-Thermal Coupling Stress

**Figure 8:**
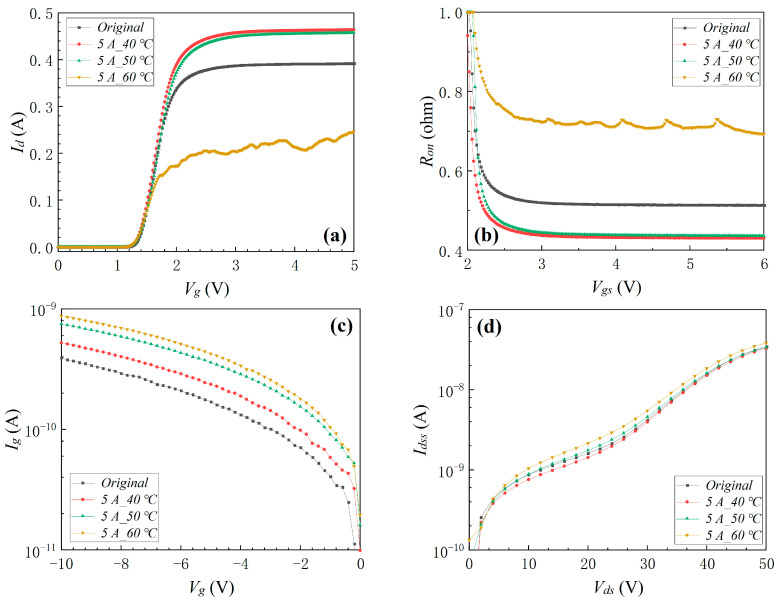
The device’s transfer characteristic (**a**), *R_on_* (**b**), *I_g_* (**c**), and *I_dss_* (**d**) before and after electro-thermal coupling stress.

In this part, the influence of the electro-thermal coupling stress on the electrical properties of top-cooled E-mode AlGaN/GaN HEMT is analyzed. The surface temperature of the device was heated to 40, 50, and 60 °C by the conducting current. The device’s transfer characteristic, *R_on_*, *I_g_*, and *I_dss_* before and after the electro-thermal coupling stress are shown in Figure 8. It can be seen that *V_th_* did not exhibit an obvious change with the increase in electro-thermal coupling stress. *R_on_* decreased by 16.2% and 15.2% when the temperature increased from the original 25 °C to 40 and 50 °C because of electro-thermal coupling stress, but it increased by 34.9% when the temperature reached 60 °C, as shown in Figure 8b. The device’s *I_g_* and *I_dss_* increased slightly from the original 25 °C to 40, 50, and 60 °C, as shown in Figure 8c,d. Additionally, when the device’s surface temperature continued to rise to 64.3 °C, the device completely failed because of the electro-thermal coupling stress. These results indicated that internal electro-thermal coupling stress was more likely to cause device failure than single environmental thermal stress.

#### 3.1.4. Thermo-Mechanical Coupling Stress

**Figure 9:**
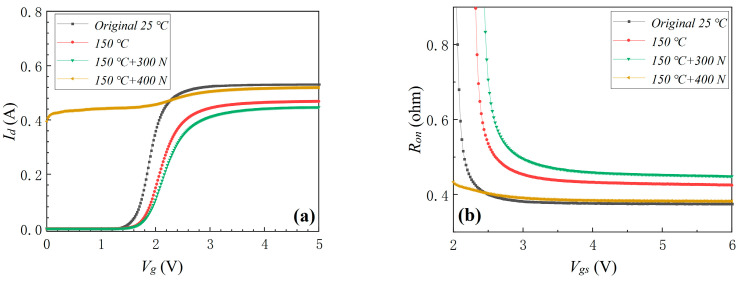

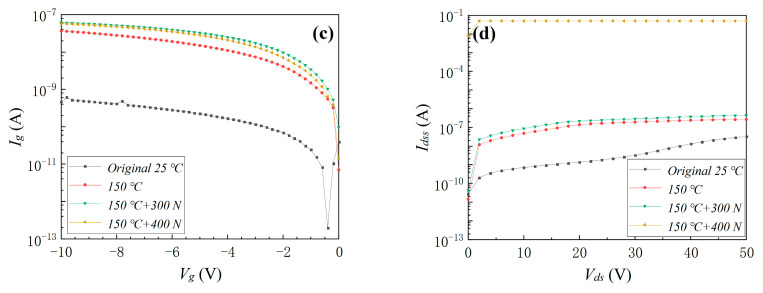
The device’s transfer characteristic (**a**), *R_on_* (**b**), *I_g_* (**c**) and *I_dss_* (**d**) before and after thermo-mechanical coupling stress.

On the basis of the environmental temperature stress results, planar pressure stresses of 300 and 400 N were applied to the device. The device’s transfer characteristic, *R_on_*, *I_g_*, and *I_dss_* before and after thermo-mechanical coupling stress are shown in Figure 9. As it can be seen in Figure 9a,b, *V_th_* did not exhibit an obvious change, and *R_on_* increased by 4.7% when the planar pressure stress increased from 0 to 300 N at the environmental temperature of 150 °C. The device’s *I_g_* and *I_dss_* increased after an applied planar pressure stress of 300 N, which was similar to the phenomena observed in Figure 4. However, after the planar pressure stress increased from 300 to 400 N at the environmental temperature of 150 °C, the device completely failed. As shown in Figure 9, *I_d_* was 0.395 A at *V_g_* = 0 V, and *I_dss_* increased to the limit value of 50 mA at *V_g_* = 2 V. The device did not fail after the mechanical planar stress of 500 N (Figure 4) and did not fail after the environmental thermal stress of 150 °C (Figure 7). When the two kinds of stresses were coupled, the device failed more quickly, indicating that mechanical planar stress coupled with environmental thermal stress aggravated the degradation of the device.

After the experiment, a new device was used for comparison with the experimental device. The two devices were detached from the PCB and their electrical parameters were tested by applying power directly to the chip pins. The electrical parameters of the new device remained normal, while those of the post-experimental device continued to fail, indicating that the contact resistance with the PCB solder joints was not a factor in the failure of the device.

#### 3.1.5. Electro-Thermo-Mechanical Coupling Stress

**Figure 10:**
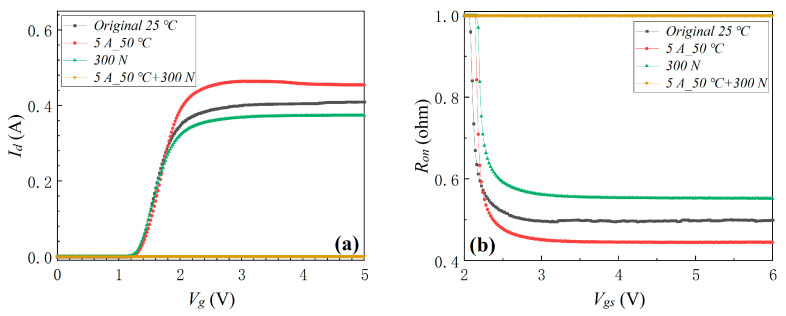

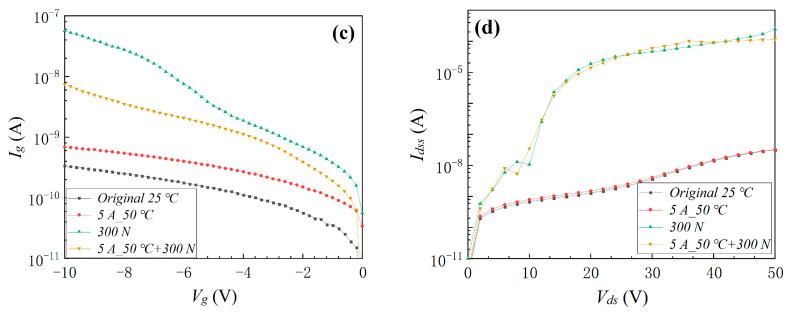
The device’s transfer characteristic (**a**), *R_on_* (**b**), *I_g_* (**c**), and *I_dss_* (**d**) before and after electro-thermo-mechanical coupling stress.

Mechanical planar pressure stress and electro-thermal coupling stress were combined to form the electro-thermo-mechanical coupling stress. The device’s transfer characteristic, *R_on_*, *I_g_*, and *I_dss_* before and after electro-thermo-mechanical coupling stress are shown in Figure 10. The results indicated that the device did not fail with the external mechanical planar stress of 300 N or the internal electro-thermal coupling stress of 50 °C individually applied to the devices. However, the device failed when the external mechanical planar stress of 300 N coupled with the internal electro-thermal coupling stress of 50 °C was applied to the device, which indicated that external mechanical planar stress coupled with internal electro-thermal coupling stress also aggravated the degradation of the device. As found in the previous electro-thermal coupling experiment, the device completely failed when the device’s surface temperature continued to increase to 64.3 °C because of the electro-thermal coupling stress. Similarly, the device completely failed when the external mechanical planar stress of 300 N was coupled with the electro-thermal coupling stress of the surface temperature rising to 48.3 °C. Comparing the above experimental results demonstrated that external mechanical planar stress coupled with internal electro-thermal coupling stress enhanced invalidation of the device.

### 3.2. Electro-Thermo-Mechanical Coupling Simulation Result and Discussion

**Figure 11:**
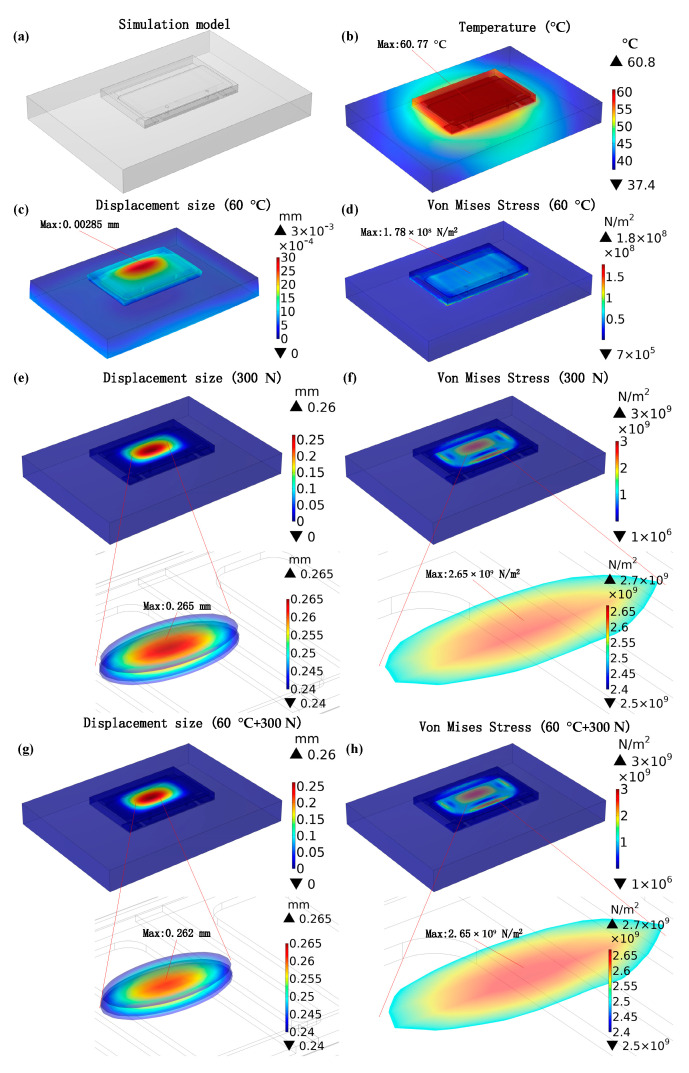
Experimental simulation model (**a**), temperature distribution (**b**), displacement size (**c**), and von Mises stress distribution (**d**) after electro-thermal coupling stress of 60 °C; displacement size (**e**) and von Mises stress distribution (**f**) after mechanical planar stress of 300 N; displacement size (**g**) and von Mises stress distribution (**h**) after electro-thermo-mechanical coupling stress.

For more in-depth investigation, finite element simulation carried with COMSOL was used to analyze the effect of electro-thermo-mechanical coupling stress on top-cooled E-mode AlGaN/GaN HEMT. The multi-field coupling simulation model of GS66508T with the PCB is shown in Figure 11a. The PCB material was FR-4 epoxy glass cloth, which has high mechanical properties. The PCB dimensions were 14 mm × 10 mm × 1.6 mm. The corner-based locations were −3.52, −2.76, and −1.6 mm. The mechanical boundary conditions placed the upper surface of the PCB as a fixed constraint because of FR-4’s high mechanical properties. Two multi-physics fields of electromagnetic heat and thermal expansion were added. The temperature distribution, displacement size, and von Mises stress distribution in an ideal GaN HEMT structure without any defects were simulated. This simulation only investigated the interaction between thermal expansion generated by electromagnetic heat and external pressure, without simulating the electrical properties of the HEMT. Figure 11b exhibits the simulated temperature distribution under electro-thermal coupling stress. The high temperature will cause structural displacement in the device due to the thermal expansion effect, as shown in Figure 11c. It can be seen that the device would be mainly thermally expanded upward and the maximum expansion displacement of the chip was 0.00285 mm, which will cause high von Mises stress inside the chip (Figure 11d). Additionally, it can be seen that the maximum von Mises stress under the electrical self-heating expansion was 1.78 × 10^8^ N/m^2^. The high von Mises stress may destroy the internal structure of the device. Figure 11e,f shows the displacement size and von Mises stress distribution for the device under an external mechanical planar stress of 300 N. The device would be squeezed and deformed downward under an external mechanical planar stress of 300 N. The maximum expansion displacement of the chip was 0.265 mm and the maximum von Mises stress inside the chip was 2.63 × 10^9^ N/m^2^.

When the external mechanical planar stress of 300 N and internal electro-thermal coupling stress of 60 °C were both applied to the device at the same time, the displacement size and von Mises stress distribution are shown in Figure 11g,h. It can be seen that the device would be squeezed and deformed under electro-thermo-mechanical coupling stress. The maximum displacement of the chip was only 0.262 mm and the maximum von Mises stress inside the chip was 2.65 × 10^9^ N/m^2^. The electro-thermo-mechanical coupling simulation results showed that the deformation due to external mechanical planar stress would collide with the thermal expansion caused by electro-thermal coupling stress, thus enhancing the internal stress field of the chip and promoting invalidation of the device compared to applying a single stress of the same size. The simulation results were consistent with the findings of the electro-thermo-mechanical coupling experiment.

## 4. Conclusions

In conclusion, this work focused on the effects of mechanical stress and carried out planar pressure, linear deformation, punctate deformation, environmental temperature, electro-thermal coupling, thermo-mechanical coupling, and electro-thermo-mechanical coupling experiments on top-cooled E-mode AlGaN/GaN HEMT (GS66508T). The experimental phenomena showed that different kinds of stress have different influence mechanisms on the device. Namely, excessive mechanical pressure/deformation stress will cause serious irrecoverable degradation of the device in *I_g_* and *I_dss_*. Additionally, electrical property degradation was enhanced by a smaller contact area between the device and the external mechanical stress source. Compared with three mechanical stresses, environmental thermal stress had a greater influence on the transfer characteristic and *R_on_* but far less influence on *I_g_* and *I_dss_*. As was expected, multiple stress coupled to the device promoted invalidation of the device compared to applying single stresses of the same size. Finally, COMSOL was used to conduct an in-depth investigation of the effects of electro-thermo-mechanical coupling stress on the device. The simulation results showed that the deformation caused by external mechanical planar stress collided with the thermal expansion caused by electro-thermal coupling stress, thus enhancing the internal stress field of the chip, which was consistent with the conclusions of the electro-thermo-mechanical coupling experiments and provided simulation support for the analysis of the experimental results. The results of the experiments and simulation demonstrated that single and coupled stresses, especially mechanical stress coupled with other stresses, will cause electrical property degradation or even irreversible damage to top-cooled E-mode AlGaN/GaN HEMT.

## Figures and Tables

**Table 1:** Model dimension, location and material.

Model Structure	Dimension (mm)	Location (mm, Corner-Based)	Material
Gate region 1	0.74 × 0.89 × 0.12	x: 0.08, y: 0.08, z: 0	Copper
Gate region2	0.74 × 0.89 × 0.12	x: 6.14, y: 0.08, z: 0	Copper
Gate region3	0.26 × 0.89 × 0.07	x: 0.82, y: 0.08, z: 0.05	Copper
Gate region4	0.26 × 0.89 × 0.07	x: 5.88, y: 0.08, z: 0.05	Copper
Source region1	4.14 × 0.89 × 0.12	x: 1.41, y: 0.08, z: 0	Copper
Source region2	0.8 × 2.4 × 0.07	x: 1.48, y: 0.97, z: 0.05	Copper
Source region3	0.8 × 2.4 × 0.07	x: 3.08, y: 0.97, z: 0.05	Copper
Source region4	0.8 × 2.4 × 0.07	x: 4.68, y: 0.97, z: 0.05	Copper
Rounding of source regions: 0.2			
Drain region1	0.3 × 2.4 × 0.07	x: 0.73, y: 1.26, z: 0.05	Copper
Drain region2	0.3 × 2.4 × 0.07	x: 2.53, y: 1.26, z: 0.05	Copper
Drain region3	0.3 × 2.4 × 0.07	x: 4.13, y: 1.26, z: 0.05	Copper
Drain region4	0.3 × 2.4 × 0.07	x: 5.93, y: 1.26, z: 0.05	Copper
Drain region5	5.71 × 0.74 × 0.12	x: 0.625, y: 3.66, z: 0	Copper
Rounding of drain regions: 0.1			
Chip region1	5 × 3 × 0.005	x: 0.98, y: 0.8, z: 0.12	GaN
Chip substrate	5 × 3 × 0.3	x: 0.98, y: 0.8, z: 0.125	Silicon
Connection column 1	R: 0.1, H: 0.54	x: 1.98, y: 0.5, z: 0	Copper
Connection column 2	R: 0.1, H: 0.54	x: 4.98, y: 0.5, z: 0	Copper
Thermal pad	6.52 × 3.09 × 0.08	x: 0.22, y: 0.08, z: 0.46	Copper
Rounding of thermal pad: 0.3			
Shell	6.96 × 4.48 × 0.54	x: 0, y: 0, z: 0	FR-4

## Data Availability

Data available on request due to restrictions, e.g., privacy or ethical.
